# Genetically predicted blood metabolites mediate the association between circulating immune cells and severe COVID-19: A Mendelian randomization study

**DOI:** 10.1097/MD.0000000000040509

**Published:** 2024-11-15

**Authors:** Ning Ai, Yan Zhang, Jing Yang, Yu Zhang, Xuejing Zhao, Huifen Feng

**Affiliations:** a Department of Gastroenterology, The Fifth Affiliated Hospital of Zhengzhou University, Zhengzhou, Henan, China.

**Keywords:** blood metabolites, circulating immune cells, Mendelian randomization, severe COVID-19

## Abstract

Investigating the causal relationship between circulating immune cells, blood metabolites, and severe COVID-19 and revealing the role of blood metabolite-mediated circulating immune cells in disease onset and progression. Genetic variation data of 731 circulating immune cells, 1400 blood metabolites, and severe COVID-19 from genome-wide association study open-access database (https://gwas.mrcieu.ac.uk) were used as instrumental variables for bidirectional and two-step Mendelian randomization analysis. The study identified 11 circulating immune cells with unidirectional causality to severe COVID-19. Two-step Mendelian randomization analysis showed 10 blood metabolites were causally associated with severe COVID-19, and blood Myristate and Citrulline to phosphate ratio mediated the association of circulating effector memory double negative % DN and CD8dim natural killer T cell % T cells, respectively, with severe COVID-19 (Myristate mediated effect ratio was 10.20%, *P* = .011; Citrulline to phosphate ratio mediated effect ratio was −9.21%, *P* = .017). This study provides genetic evidence assessing the causal relationship between circulating immune cells, blood metabolites, and severe COVID-19, elucidates the role of blood metabolite-mediated circulating immune cells in severe COVID-19 development, and offers new insights into severe COVID-19 etiology and related preventive and targeted therapeutic strategies.

## 1. Introduction

COVID-19 is an acute respiratory infectious disease caused by SARS-CoV-2 infection, which is highly contagious and characterized by high morbidity and mortality. The majority of individuals with COVID-19 exhibit mild to moderate symptoms, and some even present with asymptomatic infections, but 10% to 20% develop severe symptoms of systemic inflammation and tissue damage, acute respiratory distress syndrome, respiratory failure, coagulation, shock, and multiple organ dysfunction.^[1–5]^ As of October 2022, COVID-19 had resulted in over 6.5 million fatalities out of 620 million confirmed cases, posing an enormous threat to the global economy and public health.^[6]^ SARS-CoV-2 expresses 4 structural proteins: spike, nucleocapsid, membrane, and envelope. The spike protein mediates viral entry into cells via its interaction with angiotensin-converting enzyme 2, thereby initiating subsequent replication and transcription processes essential for infection.^[7]^

The invasion of SARS-CoV-2 into the human body triggers a series of immune-inflammatory responses, and COVID-19 induces corresponding alterations in the immune system, encompassing both innate and adaptive mechanisms.^[8]^ Despite the dynamic and complex nature of this process, the close association between COVID-19 and the immune system, as well as its cellular components, is undeniable. An autopsy study of a COVID-19 fatality demonstrated a pronounced accumulation of activated immune cells and a decreased viral load in the blood, indicating that tissue damage and organ failure could be due to immune dysregulation rather than direct viral-induced tissue injury.^[9,10]^ Considering the severity, poor prognosis, and the exceptionally high mortality rate of the disease, our study concentrates on severe COVID-19. Severe COVID-19, particularly when it progresses to severe acute respiratory distress syndrome, is believed to be driven by an overactive and dysregulated innate immune system, coupled with inadequate adaptive immune responses.^[11,12]^ It is worth noting that immune cells are divided into different types and subtypes based on the expression of different protein markers such as cluster of differentiation during normal cellular differentiation and activation, and thus exercise different immune functions. Studies have shown that the natural infection of the SARS-CoV-2 pathogen or related vaccines can induce specific serological immune responses in the innate and adaptive immune systems, which are specific to immunogenic epitopes of the pathogen.^[13,14]^ Monocytes and macrophages are antigen presenting cells with the phagocytic function of bone marrow. Under normal circumstances, monocytes exist in the peripheral circulation and are recruited by stimulated tissues to exert chemotactic functions, and macrophages resident in alveolar tissue are also called alveolar macrophages. Alveolar macrophages serve as resident macrophages in alveolar tissue. In response to SARS-CoV-2 infection, these 2 cells act as sentinel response cells that prioritize responses such as the activation of inflammatory vesicles via relevant molecules to trigger the release of inflammatory factors.^[15,16]^ Proper immune responses facilitate virus clearance, but aberrant monocyte and macrophage activation or dysregulation in response to SARS-CoV-2 can intensify tissue damage and prolong recovery, potentially contributing to severe COVID-19.^[17,18]^ An excess of dysfunctional monocytes, which exhibit reduced antiviral capacity, and inflammatory monocytes, which promote systemic inflammation and alter vascular permeability through up-regulation of cell adhesion molecules, were observed in severe COVID-19 patients.^[8,9,13,19–22]^ The simultaneous replacement of resident alveolar macrophages by macrophages derived from inflammatory monocytes has been documented.^[23,24]^ Within innate immunity, neutrophil dynamics are crucial to the progression of severe COVID-19. Evidence indicates a pronounced phenomenon of emergency myelopoiesis, whereby the bone marrow mobilizes numerous immature cells in response to acute pathological injury to the organism, was observed in severe COVID-19.^[25]^ Among them, numerous immature neutrophils expressing the inhibitory molecule programmed death ligand 1 (PD-L1) were observed to be activated.^[25–28]^ The abnormal proliferation of bone marrow, resulting from certain pathological conditions, leads to an increase in the number of immature myeloid cells. These cells predominantly suppress T cell proliferation and induce lymphocyte apoptosis, and are collectively termed myeloid-derived suppressor cells (MDSCs). MDSC mainly contains 2 subsets, granulocytic or polymorphonuclear MDSCs (PMN-MDSCs) and monocytic MDSCs (M-MDSCs). The specific molecular markers on the surface of MDSC indicate the corresponding phenotypic changes. PMN-MDSC is most often described as CD11b + CD33dim HLA-DR− CD14− CD15 + CD66b+, whereas M-MDSC is mainly defined as CD11b + CD33hi HLA-DR− CD14+ CD15−.^[29]^ However, the phenotype of immature neutrophils in severe COVID-19 is also changed during the pathological process. Research indicates that immature neutrophil surface markers and gene expression profiles in the peripheral blood of severe COVID-19 patients may bear similarities to those of PMN-MDSCs. However, the exact correlation remains unclear. Nonetheless, both may exhibit similar immunosuppressive functions in severe COVID-19.^[9]^ While mature-activated neutrophils are found in both mild and severe COVID-19, those in only severe COVID-19 could express PD-L1.^[9]^ In conclusion, the excessive activation of neutrophils in severe COVID-19 is significantly linked to immunosuppression. Despite evidence of impaired oxidative burst capacity in these neutrophils, the presence of neutrophil extracellular traps which may be regulated by interleukin-6, and their excessive activation in severe COVID-19 implicate a potential role in amplifying the inflammatory cascade and activating platelets to promote thrombosis.^[13,30–32]^ Studies have shown that SARS-CoV-2 infection specifically induces an immune response in B cells. For example, vaccines have been successfully developed on the basis of the principle that SARS-CoV-2-specific B cells primarily produce and act on IgG antibodies against the spike protein. Furthermore, in vitro studies suggest that SARS-CoV-2 IgA1 and IgG3 may have protective neutralizing effects in SARS-CoV-2 infection.^[33,34]^ Studies indicate that SARS-CoV-2-specific T cell responses during the acute and convalescent phases of COVID-19 promote the progression of pneumonia symptoms towards milder levels, implying that specific T cell responses in adaptive immunity may contribute to virus clearance and prevent reinfection.^[35,36]^ Compared with mild COVID-19, helper CD4 + T cells and cytotoxic CD8 + T cells with cytolytic function were significantly reduced in peripheral vasculature in patients with severe COVID-19.^[37,38]^ PD-L1, together with PD-1, can be regarded as a checkpoint, marking the degree of immunosuppression of T cells. The expression of PD-1 on the surface of CD4 + T cells and CD8 + T cells in peripheral blood is significantly increased, indicating the depletion and failure of T cells.^[39]^ Signaling between CD4 and CD8 T cells and antigen presenting cells and B cells expressing major histocompatibility complex (MHC) type II molecules is essential for health.^[13]^ In addition, double negative DN B cells and double negative DN T cells have multiple phenotypes and their current understanding is not comprehensive. Therefore, the roles of DN B cells and DN T cells deserve further research.^[13,40]^ This was further explored in our genome-wide association study (GWAS) study.

Immune cells are crucial in the transition from SARS-CoV-2 infection to severe COVID-19, and potentially to a fatal outcome. Investigating the roles and molecular mechanisms of potential immune cells in this process is essential for understanding the pathogenesis of severe COVID-19 and for identifying prognostic biomarkers and therapeutic targets. Prior observational and cohort studies have encountered issues with reverse causality, making it unclear whether specific immune cells influence severe COVID-19 or if severe COVID-19 impacts these cells. Mendelian randomization (MR) analysis, an epidemiological approach grounded in GWAS, utilizes genetic variation and single nucleotide polymorphisms (SNPs) as instrumental variables (IVs) to ascertain the relationship with outcomes, mitigating the effects of confounding factors.^[41]^ Observational studies demonstrate a correlation between dysregulated blood metabolites, such as amino acids, fatty acids, and lipids, and the severity of COVID-19.^[42–45]^ For example, tryptophan is an essential amino acid regulated by enzyme indoleamine 2,3-dioxygenase-1 (IDO-1) or indoleamine 2,3-dioxygenase-2 (IDO-2), which ultimately produces kynurenine. Relevant studies have shown that tryptophan degradation products, which may lead to cell death, are widely accumulated in the lungs in the autopsy cohort of COVID-19 patients, which is reduced tryptophan and increased L-kynurenine. Markers of apoptosis and severe cellular stress were associated with the IDO-2 expression in large areas of lung tissue, which may confirm the expression of IDO in COVID-19. Early initiation of the kynureine/aryl-hydrocarbon receptor/IDO-2 axis as a positive feedback pathway is thought to contribute to severe COVID-19 pathology.^[13,46,47]^ At the same time, studies have shown that IDO can promote the differentiation of macrophages in the anti-inflammatory M2 direction, which reveals a possible response to SARS-CoV-2 infection of macrophages.^[48]^ The involvement of immune cells in diseases involves multiple metabolic pathways; however, how blood metabolites participate in the influence of circulating immune cells on the production of severe COVID-19 remains unclear. Elucidating the potential role of blood metabolites among circulating immune cells in severe COVID-19 is critical for constructing a holistic immune-metabolic network associated with the disease. Consequently, we incorporated blood metabolites as a mediator index in our analysis, employing two-step MR analysis to explore the intermediary effects of these metabolites.

## 2. Methods

### 2.1. Study design

This study-related factor encompassed 731 circulating immune cells, 1400 blood metabolites, and severe COVID-19. We aimed to explore their causal relationship through a two-sample, bidirectional, and two-step MR analysis. The following 3 points should be emphasized for two-step MR analysis: assumption of no confounding: including no confounding between variables and no additional confounding; no interaction between variables and mediation variables; no reverse causal effect between outcome variables and variables.

### 2.2. Data sources

#### 2.2.1. Source of circulating immune cells data

The genetic data of the GWAS of immune cells used in this study came from a survey of the genetic characteristics of 731 immune cells with a comprehensive cell phenotype.^[49]^ The GWAS statistical data of each immune cell can be obtained from the authoritative genome-wide association research websites of the GWAS Catalog (https://www.ebi.ac.uk/gwas) and the IEU Open GWAS Project (https://gwas.mrcieu.ac.uk). We included various circulating immune cells such as B lymphocytes, T lymphocytes, monocytes, bone marrow cells, dendritic cells, natural killer (NK) cells, and regulatory T cells (Treg).

#### 2.2.2. Source of blood metabolites data

The blood metabolites GWAS data originated from a study employing ultra-performance liquid chromatography-tandem mass spectrometry, measuring the ratio of 1091 blood metabolites and 309 blood metabolites in human plasma.^[50]^ The GWAS statistics corresponding to each blood metabolite are accessible in the online GWAS Catalog database.

#### 2.2.3. Source of severe COVID-19 data

GWAS data of severe COVID-19 were sourced from ebi-a-GCST010775 dataset transferred from EBI database to IEU Open GWAS project website. Ebi-a-GCST010775 encompassed 9201,012 SNPs of severe COVID-19 patients and mild non hospitalized COVID-19 patients from European ancestry.

### 2.3. Selection of IVs

#### 2.3.1. Association analysis

First, we acquired the GWAS data for each research factor. Typically, the factors involved in MR are divided into complex phenotypes affected by polygenic and polybiological pathways, and molecular traits as genetic research. The former typically possesses a multitude of SNPs directly associated with it, while the latter is characterized by a limited number of SNPs reflecting genetic variation. Consequently, in our association analysis, we employed a *P*-value < 1 × 10^−5^ for selecting SNPs associated with circulating immune cells and blood metabolites as exposure variables. For the reverse MR analysis, with severe COVID-19 as the exposure variable, we set the correlation threshold at a *P*-value of 5 × 10^−6^.

#### 2.3.2. Removal of linkage disequilibrium

Remove SNPs that are prone to linked inheritance due to their genomic proximity, using a criterion of kb > 10,000 and *R*^2^ < 0.001 to filter out SNPs influenced by linkage disequilibrium. Preserve the SNP with the minimal *P*-value linked to the exposure variable, hereby ensuring the independence of the selected SNPs from one another.

#### 2.3.3. Evaluating weak IV bias

We determined the *R*^2^ value for an individual SNP and calculated the F-test value to assess weak IV bias. *R*^2^ = 2 × beta^2^ × eaf × (1 − eaf), where the beta value is the effect value of the SNP, the eaf value is the effect size of the SNP, *R*^2^ is the degree of single IV exposed interpretation. F = ((N − k − 1)/k) × (*R*^2^/(1 − *R*^2^)), N represents the number of samples in the GWAS study for this factor and k represents the number of IV, since we calculated the F-test value of a single SNP, k = 1. Retain SNP with the F-test value > 10 and remove SNP with weak tool variable bias.^[51]^

#### 2.3.4. Confusing factor

MR is predicated on the assumption that IVs should not affect the outcome through other traits explicitly linked to the outcome variables or directly influence the outcome variables themselves. Given that immune cells and blood metabolites, regulated by relatively singular genes and pathways, a mediate two-step MR analysis process without reliance on the hybridization hypothesis, therefore, our MR study did not eliminate the hybridization factors of SNPs. Utilize the PhenoScanner website to identify genome-wide traits significantly associated with the corresponding SNPs (*P* < 1 × 10^-5^), and exclude those SNPs that are directly related to the outcome factors. To minimize the impact of horizontal pleiotropy on causality, we performed a pleiotropy test as part of our sensitivity analysis to explore the potential effects of confounding factors.

#### 2.3.5. Extraction of SNPs from GWAS data of outcome factors

SNPs extracted from the outcome factor GWAS data are replaced with proxy SNPs (*R*^2^ > 0.9 when the proxy SNP is available) in the case that SNPs cannot be extracted from the dataset due to interpolation differences. Combine the exposure and outcome SNPs, excluding those that are directly outcome-associated, align the allele loci to match the effects of exposure and outcome SNPs, and exclude palindromic SNPs with allele frequencies ranging from 0.42 to 0.58.^[52]^ The selected SNPs ultimately served as the IVs in the MR analysis.

### 2.4. MR analysis

Throughout the study, we primarily utilized the inverse-variance weighted (IVW) method within a random-effects model to assess the causal effects of exposure on outcomes in MR analysis. Additionally, we employed the weighted median, MR-Egger, simple mode, weighted mode methods as supplementary analyses. The effects of exposure on outcomes or mediators were expressed as odds ratios (OR) and 95% confidence intervals (CI).

We respectively conducted the two-sample MR analysis of circulating immune cells and blood metabolites and severe COVID-19 to explore the causal relationship. We established *P*-value = .01 as the threshold for determining the significance of causal effects. The “beta-all” value obtained by the initial analysis serves as the overall effect for subsequent mediation MR analysis.

To investigate the potential mediation of blood metabolites in the causal relationship between circulating immune cells and severe COVID-19 pneumonia, we used two-step MR analysis to link “circulating immune cells, blood metabolites, and severe COVID-19 pneumonia.” Summary of the circulating immune cells and blood metabolites that have significant causal effects on severe COVID-19. In the first step of the two-step MR analysis with circulating immune cells as exposure factors and blood metabolites as outcome factors, *P*-value < .05 was considered to have significant statistical differences, and the resulting “beta-1” value was utilized to estimate the mediating effect. The second step of analysis is based on the blood metabolites identified and the severe COVID-19 in the first step. It is essential to note that if the IV utilized in the subsequent step has any overlap with that from the initial step, such IV must be excluded, and the derived “beta-2” value is then employed to calculate the mediating effect. The total effect of circulating immune cells is apportioned into a direct effect, where immune cells influence the outcome without mediation, and an indirect effect mediated by blood metabolites. Calculate the mediation effect using “beta-1 × beta-2,” calculate the percentage mediated by the mediation effect using “beta-1 × beta-2/ beta all,” and use the Delta method to calculate the 95% CI and *P*-test value of the mediation effect mediation.^[53]^

The circulating immune cells identified through the two-step MR analysis served as the outcome variable, and severe COVID-19 was considered as the exposure variable in the reverse MR analysis to investigate the reverse causal relationship between them. Based on the mediated MR hypothesis and existing literature, the outcome and exposure variables in the mediation MR analysis must satisfy the absence of reverse causality, adopting a *P*-value = .05 for the evaluation threshold.

### 2.5. Sensitivity analysis

Horizontal pleiotropy is assessed by estimating the deviation of the MR-Egger intercept, which serves as a measure of the average pleiotropic effect across genetic variants. If the intercept significantly deviates from 0 with a *P*-value < .05, the presence of horizontal pleiotropy is indicated, and this scenario is disallowed.^[54]^ Heterogeneity was assessed by calculating *P*-values derived from the Cochran Q statistic using both IVW and MR-Egger methods, and a Q-pval < .05 was considered indicative of heterogeneity.^[55]^ The MR pleiotropy residual sum and outlier method is utilized for the detection of horizontal pleiotropy and the identification of biased SNPs. If an SNP exhibits horizontal pleiotropy or has a *P*-value < .05, the MR analysis should be reiterated.^[56]^ The leave-one-out analysis assesses the robustness of the MR analysis by sequentially removing each SNP.^[57]^

### 2.6. Statistical processing

Implement all statistical analysis and data visualization through R software packages such as R version 4.3.2 and TwoSampleMR and others.

## 3. Results

### 3.1. Bidirectional causal relationship between circulating immune cells and severe COVID-19

**Figure 1.**
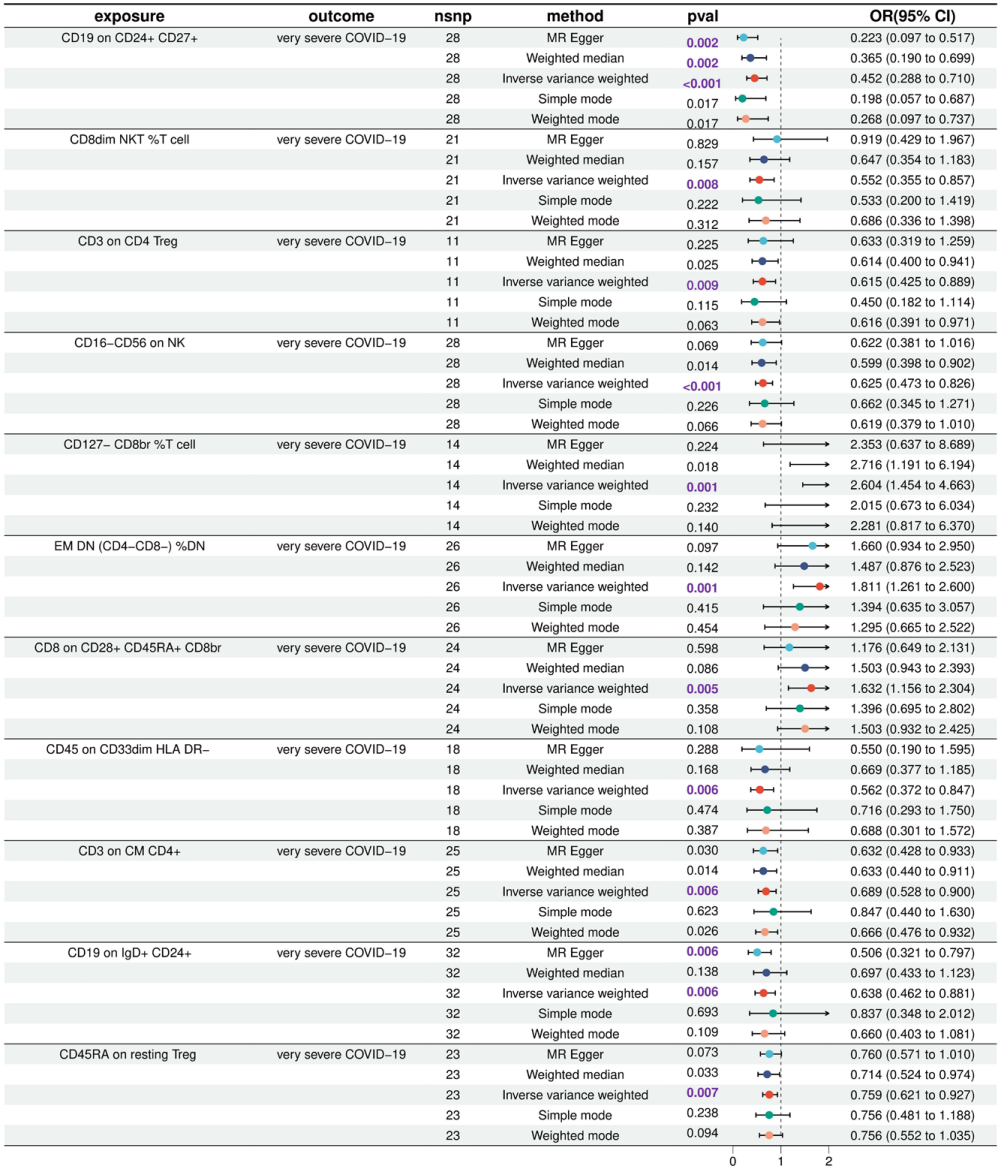
Mendelian randomization results of potential and unidirectional causal relationship between 11 circulating immune cells and severe COVID-19.

Genetic variation in exploring the bidirectional causal relationship between circulating immune cells and severe COVID-19 predicted a potential causal relationship between 12 circulating immune cells and severe COVID-19. The respective IVW results for these were as follows: CD127- CD8br % T cell (OR = 2.60; 95% CI = 1.45–4.66; *P* = .001), effector memory double negative (EM DN) % DN (OR = 1.81; 95% CI = 1.26–2.60; *P* = .001), CD8 on CD28 + CD45RA + CD8br (OR = 1.63; 95% CI = 1.16–2.30; *P* = .005) was positively correlated with severe COVID-19, indicating that the increase of leukocyte differentiation antigen abundance in each of them would lead to the risk of severe COVID-19; CD16- CD56 on NK (OR = 0.63; 95% CI = 0.47–0.83; *P* < .001), CD19 on CD24 + CD27 + (OR = 0.45; 95% CI = 0.29–0.71; *P* < .001), CD45 on CD33dim HLA-DR- (OR = 0.56; 95% CI = 0.37–0.85; *P* = .006), CD3 on central memory (CM) CD4 + (OR = 0.69; 95% CI = 0.53–0.90; *P* = .006), CD19 on IgD + CD24 + (OR = 0.64; 95% CI = 0.46–0.88; *P* = .006), CD45RA on resting Treg (OR = 0.76; 95% CI = 0.62–0.93; *P* = .007), CD8dim natural killer T cell (NKT) % T cell (OR = 0.55; 95% CI = 0.35–0.86; *P* = .008), CD3 on CD4 Treg (OR = 0.61; 95% CI = 0.42–0.89; *P* = .009), transitional % B cell (OR = 0.37; 95% CI = 0.19–0.70; *P* = .003) was negatively correlated with severe COVID-19, indicating that the increase of leukocyte differentiation antigen abundance in each of them would reduce the risk of severe COVID-19. Severe COVID-19 had a potential reverse causality to transitional % B cell (OR = 1.019; 95% CI = 1.004–1.035; *P* = .012). So we removed the transitional % B cell from the circulating immune cells in the subsequent MR. Among the remaining 11 circulating immune cells, CD19 on CD24 + CD27+, transitional % B cells, CD19 on IgD + CD24 + belong to B cells; CD16-CD56 on NK, CD8dim NKT % T cell belong to NK cells; CD127- CD8br % T cells, CD8 on CD28 + CD45RA + CD8br, CD45RA on resting Treg, CD3 on CD4 Treg belong to Treg cells; EM DN % DN, CD3 on CM CD4 + belong to T cells; CD45 on CD33dim HLA-DR- belongs to myeloid cells. Ultimately, we revealed the potential and unidirectional causal relationship between 11 kinds of circulating immune cells and severe COVID-19 (Fig. 1).

### 3.2. Mediation MR analysis of potential blood metabolites

**Figure 2.**
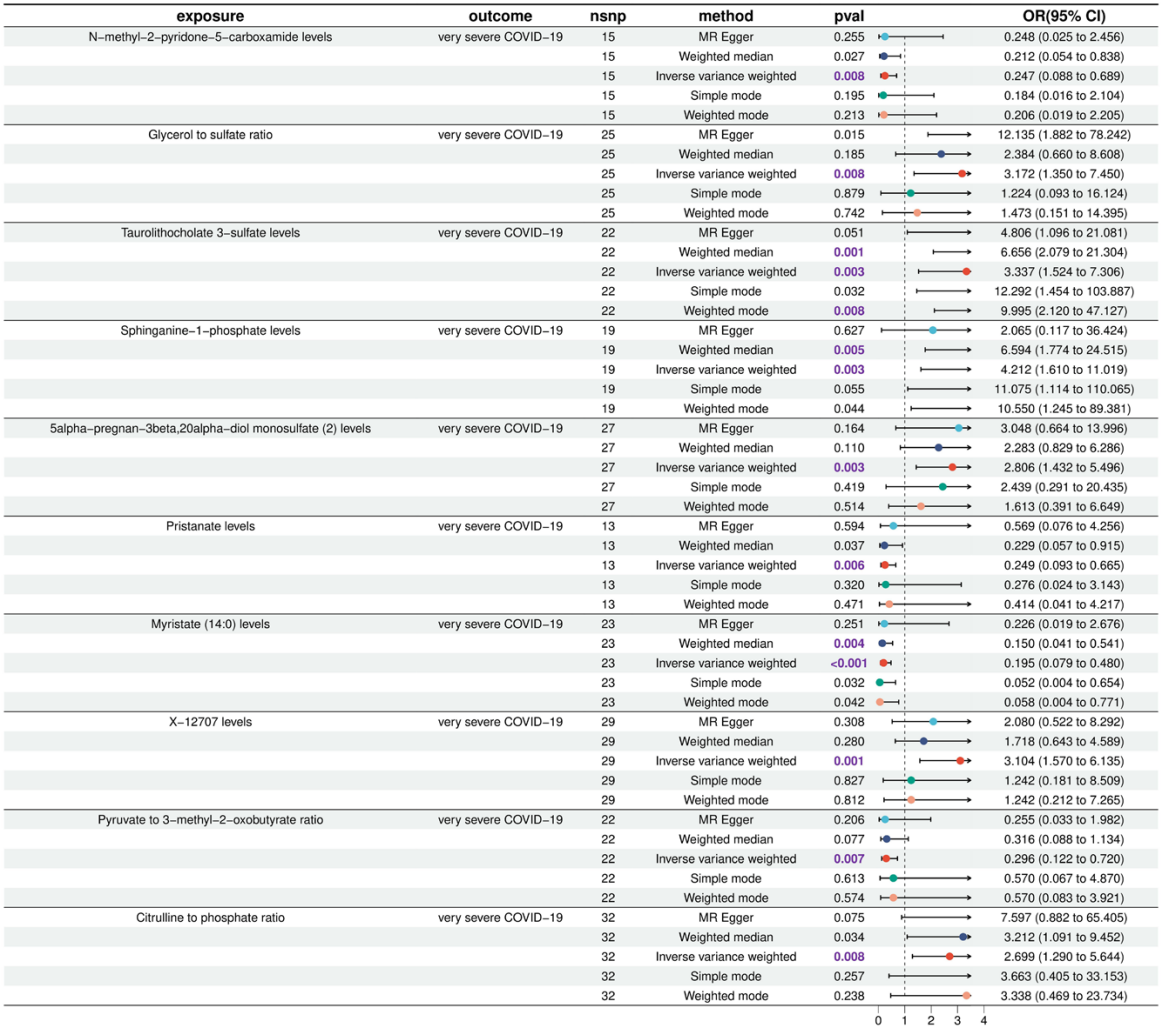
Mendelian randomization results of potential causal relationship between 10 blood metabolites and severe COVID-19.

**Figure 3.**
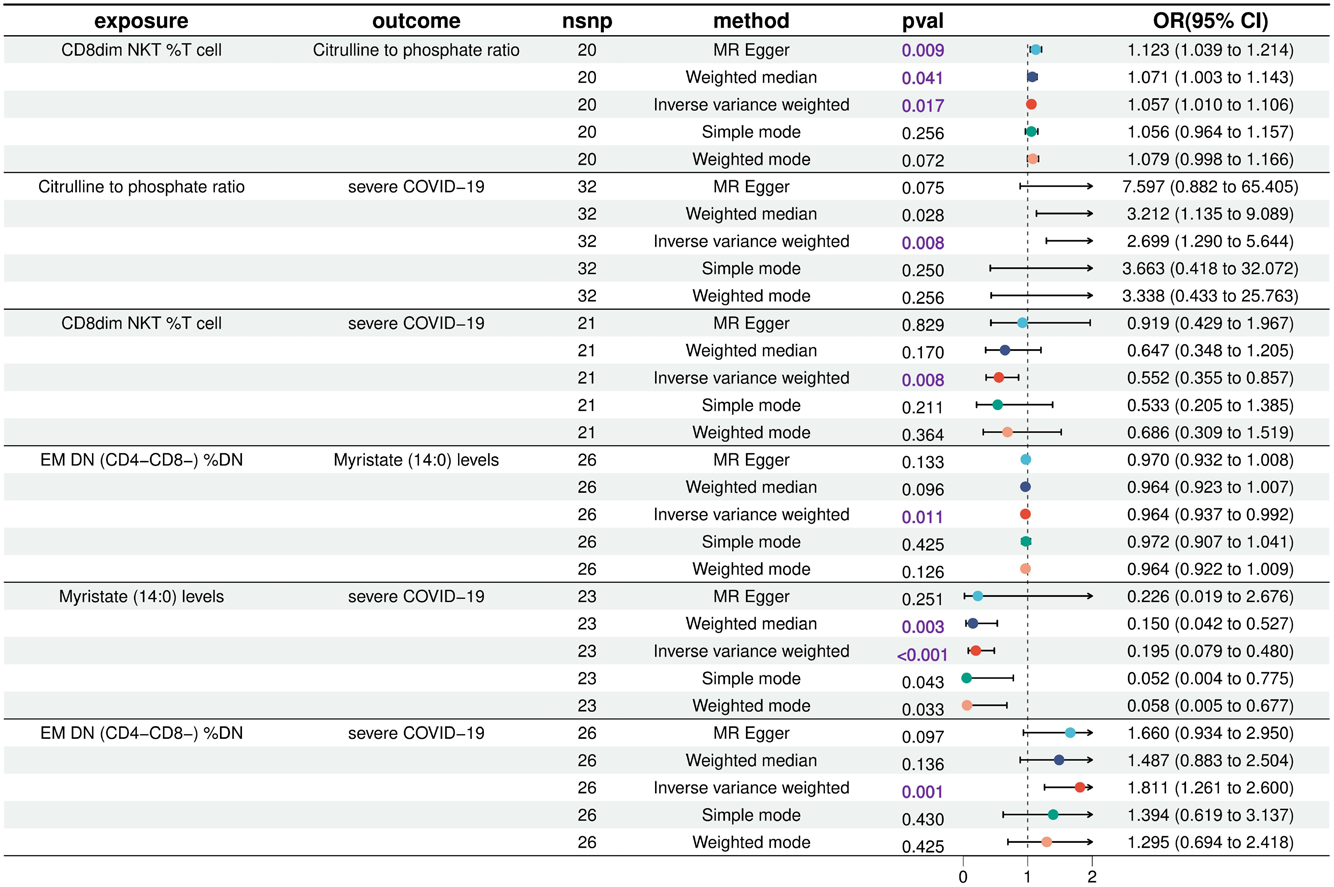
Two-step Mendelian randomization analyses show causal effects between circulating immune cells, blood metabolites, and severe COVID-19.

The two-step MR demonstrated a causal relationship between the 10 blood metabolites and severe COVID-19. The following metabolites showed significant associations: Sphinganine-1-phosphate levels (OR = 4.21; 95% CI = 1.61–11.02; *P* = .003), Taurolithocholate 3-sulfate levels (OR = 3.37; 95% CI = 1.52–7.31; *P* = .003), glycerol to sulfate ratio (OR = 3.17; 95% CI = 1.35–7.45; *P* = .008), X-12707 level (OR = 3.10; 95% CI = 1.57–6.13; *P* = .001), 5-alpha-pregnan-3beta (OR = 2.81; 95% CI = 1.43–5.50; *P* = .003), Citrulline to phosphate ratio (OR = 2.70; 95% CI = 1.29–5.64; *P* = .008) were positively correlated with severe COVID-19, Pyruvate to 3-methyl-2-oxobutyrate ratio (OR = 0.30; 95% CI = 0.12–0.72; *P* = .007), Pristanate levels (OR = 0.25; 95% CI = 0.09–0.66; *P* = .006), N-methyl-2-pyridone-5-carboxamide levels (OR = 0.25; 95% CI = 0.09–0.69; *P* = .008), Myristate (14:0) levels (OR = 0.19; 95% CI = 0.08–0.48; *P* = .004) were negatively correlated with severe COVID-19 (Fig. 2). There are 2 groups of significant causal relationships between 11 circulating immune cells that have the causal relationship with severe COVID-19 and the above 10 blood metabolites: CD8dim NKT % T cell was positively correlated with Citrulline to phosphate ratio, EM DN % DN was negatively correlated with Myristate (14:0) levels. Citrulline to phosphate ratio and Myristate (14:0) levels as mediators between severe COVID-19 and circulating immune cells, we believe that an increase in CD8dim NKT % T cells may elevate the Citrulline to phosphate ratio and thus increase the risk of severe COVID-19; a higher level of EM DN % DN in blood will result in a decrease in the concentration of Myristate (14:0), which in turn will heighten the risk of severe COVID-19 (Fig. 3). Our study showed that the Citrulline to phosphate ratio accounted for −9.21% of the reduction of CD8dim NKT % T cell risk associated with severe COVID-19, and the fluctuation range was −1.62% to −16.80%, *P* = .017; Myristate (14:0) levels accounted for 10.20% of the reduction of EM DN % DN risk associated with severe COVID-19, and the fluctuation range was 2.33% to 18.07%, *P* = .011 (Table 1). These results preliminarily suggest that circulating EM DN (CD4- CD8-) % DN reduces the inhibitory effect of Myristate (14:0) on severe COVID-19 by down-regulating the level of Myristate (14:0) in blood, thereby increasing the risk of severe COVID-19. However, Citrulline to phosphate ratio as a serum metabolite had a masking effect, the mediation effect of display and CD8dim NKT % T cell and severe COVID-19 between the total effect in the opposite direction.

**Table 1:** The mediation effect of Citrulline to phosphate ratio and Myristate (14:0) levels as mediators, respectively.

Immune cell	Metabolite	Outcome	Mediated effect	Mediated proportion	*P*-value
CD8dim NKT %T cell	Citrulline to phosphate ratio	Very severe COVID-19	0.0548 (0.00966 to 0.0999)	−9.21% (−1.62% to −16.8%)	0.017
EM DN (CD4^-^CD8^-^) %DN	Myristate (14:0) levels	Very severe COVID-19	0.0606 (0.0138 to 0.107)	10.2% (2.33 to 18.1%)	0.011

### 3.3. Sensitivity analysis

The sensitivity analysis concluded that none of the aforementioned causal relationships exhibited horizontal pleiotropy or heterogeneity. Furthermore, the MR pleiotropy residual sum and outlier and leave-one-out analyses indicated that no additional treatment for IVs was required.

## 4. Discussion

In this study, we conducted MR analysis using large-scale genetic data to explore the causal relationship between 731 circulating immune cells, 1400 blood metabolites, and severe COVID-19. Utilizing stringent inclusion criteria and sensitivity analyses, we identified 11 distinct circulating immune cells and 10 blood metabolites as potential causal relationships linked to severe COVID-19. Two groups of causal relationships were observed between the 11 circulating immune cells and 10 blood metabolites, with 2-specific blood metabolites potentially serving as mediators within the implicated immune pathways.

T lymphocytes are part of the acquired immune system and are crucial in carrying out and regulating both humoral and cell-mediated immune responses. The majority of T cells mature in the thymus, a specialized environment that facilitates the development of lymphocyte precursors into functional T cells.^[58]^ Lymphocyte precursors is transferred from the bone marrow to the thymus and transformed into immature DN cells in there, which undergo 4 stages of differentiation.^[59]^ The DN3 stage rearranges 1 of the 2 TCRβ chain gene loci and expresses the pre-T cell receptor, then the enters DN4 stage, which is followed by the immature CD8 + intermediate single-positive (SP) cell stage. The subsequent rearrangement of the TCRα chain gene results in the formation of the mature TCRαβ^+^ receptor, with the expression of CD4 + marking the transition of T cells to the double-positive (DP) phase of CD4 + CD8+.^[60,61]^ αβTCR on DP cells needs to engage with MHC I/II molecules on the surface of the thymic stromal cells, and DP cells with suitable affinity are selected to differentiate into SP T cells. DP cells binding to MHC I molecules differentiate into CD8 + T cells, while those binding to MHC II molecules become CD4 + T cells. DP cells that failed to bind to the thymic stromal cells surface MHC I/II or exhibited excessive affinity undergo apoptosis. After the positive selection of SP cells for negative selection and thymic dendritic cells, macrophages, and others, the surface of antigen peptide complex interactions. High-affinity binding SP cells undergo apoptosis as autoreactive T cells, while a small proportion of these cells may differentiate into regulatory T cells, nonbinding SP cells mature and proceed to the peripheral immune system.^[62–65]^ Different from the above mainstream process of differentiation and development of DP cells, these cells can also differentiate into NKT cells. NKT cells exhibit both the NK cell surface marker CD56 and the T cell surface marker TCRαβ-CD3 complex. NKT cells are categorized into 2 primary subtypes based on their reliance on CD1d expression by thymic cortical cells: CD1d-dependent NKT cells, which recognize lipid antigen TCR presented, and CD1d-independent NKT cells with distinct TCR repertoires. CD4 + CD8- and CD4- CD8- NKT cells mature into CD1d-dependent NKT cells, whereas CD8 + CD4- NKT cells develop into CD1d-independent NKT cells.^[66]^ Part of the DN3 stage differentiates into TCRγδ+T cells, belonging to a distinct lineage characterized by diverse phenotypes and unique traits.^[67]^ Approximately 95% of T cells in humans and mice express TCRαβ^+^, and the remaining 5% express TCRγδ^+^.^[68]^

DNT cells contain varying proportions of TCRαβ+ T cells and TCRγδ+ T cells. Relevant studies have shown that human peripheral DNT cells express polyclonal TCRαβ+,^[69]^ so we primarily concentrate on TCRαβ+ DNT cells. TCRαβ+ DNT cells comprise 1% to 3% of the mature peripheral CD3 + T cells.^[69,70]^ Unlike immature DN cells that participate in thymic T cell development and differentiation, mature TCRαβ+ DNT are found in peripheral blood, secondary lymphoid tissues, and diverse organs, including the intestine, liver, lung, skin, and reproductive tract, in both human and murine models.^[71]^ Existing studies have shown that the sources of mature TCRαβ+ DNT cells are categorized based on thymic dependency, and DNT cells are derived from the DP cell stage in the thymus or mature CD8 + T cells or even CD4 + T cells in the periphery.^[71,72]^ Exists in all kinds of organizations in which the TCRαβ+ DNT cells known as tissue reside DNT cells, its involved in bacterial and viral infections, exhibiting distinct protective or pathogenic roles depending on the infection type, and displaying varied phenotypes under different physiological and pathological conditions.^[59,71]^ EM DN cells refer to TCRαβ^+^ DNT cells in peripheral blood that exhibit effect-memory phenotype, and relevant studies have shown that the surface of these cells may be characterized by the expression of CD3 + CCR7 − CD45RA−.^[73]^ However, the current study of EM DN T cells in peripheral blood is still in the exploratory stage, and further analysis of markers may be required in the future to determine the phenotype of the cell and explore the association of the cell with DN1, DN2, DN3, and DN4 T cells. Recent studies indicate a notable rise in the proportion of CD45RA − effector DNT cells in response to TCR complex stimulation, suggesting a propensity for activated DNT cells to differentiate into the effector memory T cell phenotype.^[73,74]^ So EM DN % DN can be regarded as reflecting the human body as a current or some possible pathogen infection status indicator. Myristate, a carbon 14 linear chains saturated fatty acid in the blood, can be enzymatically linked to N-terminal glycine residues by N-myristoacyl transferase to produce a protein modification called N-myristoylation.^[75]^ Our mediation MR analysis demonstrated that an increase in EM DN % DN, indicative of a heightened risk of infection by potential pathogens, is associated with an increased risk of severe COVID-19. This elevated risk of pathogen infection results in a reduction of blood Myristate levels, consequently diminishing its protective role against severe COVID-19. Myristate, as a blood intermediary metabolite, mediated 10.20% of the causal effect. Corroborating evidence from related studies supports the findings of our mediation MR analysis: During SARS-CoV-2 infection, the cyclic GMP-AMP synthase-stimulator of interferon genes (cGAS-STING) axis, which acts as innate immune sensing and signaling in response to SARS-CoV-2, will be activated. Activation of the cGAS-STING axis may increase the risk of endothelial cell death by inducing macrophages in areas of endothelial inflammation that are affected by multiple cytokines alongside interleukin-6 (which is a neutrophil chemoattractant).^[76,77]^ Furthermore, the detection of cGAS-STING activity in lung samples from severe COVID-19 patients offers a potential explanation for the observed vascular injury and coagulopathy in those severe patients.^[78]^ Studies indicate a decrease in blood Myristate concentration following pathogen invasion. Myristate promotes STING-dependent autophagy degradation of interferon gene-stimulating factor/tank-binding kinase 1 complex by enhancing N-myristoylation of GTase ADP-ribosylation factor 1, thereby weakening the type I interferon (IFN) response induced by the cGAS-STING axis.^[75]^ In addition, our results suggest an elevated risk of severe COVID-19 in individuals coinfected with SARS-CoV-2 and other pathogens. This aligns with empirical research, demonstrating a significantly heightened risk of severe disease and mortality when SARS-CoV-2 infection is accompanied by conditions such as bacteremia, mycobacterial infection, influenza, and leptospirosis.^[79–82]^ Given that Myristate can be dietary supplemented, it is suggested that Myristate supplementation mitigates the risk of severe COVID-19 during coinfections.^[83]^ This also presents novel opportunities for the therapeutic targeting of STING inhibitors in the treatment of co-occurring infections alongside COVID-19. In addition, relevant studies have shown that DNB cells have 4 phenotypes (DN1, DN2, DN3, and DN4) based on specific differentiation clusters, chemokines, and antibodies on the cell surface. DN2 B cells and DN3 B cells have shown a certain correlation in severe COVID-19.^[13]^ DNB cells and DNT cells have been implicated in autoimmune diseases.^[13,40]^ At present, the function of DNB cells and DNT cells in severe COVID-19 is still full of unknown, and it is expected to further investigate and study DNB and DNT cells by GWAS analysis in the future.

CD8dim NKT cells refer to NKT cells with weak CD8 positive on the cell surface and in essence belong to CD8 + CD4- NKT cells that co-express T cell and NK cell surface markers and require MHC I molecules for development but are independent of CD1d.^[84]^ CD8 + NKT cells constitute <1% of the T cell population, and current research on these cells is limited, with numerous aspects remaining unknown. The current study suggests that these cells may possess features such as killing antigen-presenting dendritic cells in an antigen-specific manner and thereby inhibiting immune responses; killing tumor cells and MDSC-carrying tumor antigens within the tumor microenvironment in an antigen-specific manner, exhibiting both NK-like and CTL-like antitumor activities.^[84,85]^ Macrophages can be divided into 3 different phenotypes by polarization: M0 (nonactivated), M1 (proinflammatory), and M2 (anti-inflammatory). However, these phenotypes are still not defined by the CD nomenclature.^[86,87]^ During nitric oxide synthase (NOS) expression activity, L-arginine and nicotinamide adenine dinucleotide phosphate are metabolized to nitric oxide and the byproduct Citrulline, indicating that the plasma Citrulline to phosphate ratio may reflect NOS expression activity.^[88,89]^ Our mediation MR analysis showed that: elevated levels of CD8dim NKT % T cells were associated with a reduced risk of severe COVID-19 and the mediating effect, through the Citrulline to phosphate ratio in blood indicative of NOS expression activity, was in the opposite direction to the total effect, suggesting that an increase in CD8dim NKT % T cells can enhance active NOS expression, thereby amplifying NOS protective effect against the risk of severe COVID-19. This implies that the heightened NOS expression may partially mitigate the inhibitory impact of CD8dim NKT cells on the progression of severe COVID-19. The ratio of Citrulline to phosphate, as a blood intermediary metabolite that exerted a masking effect, mediated −9.21% of the causal effect. Empirical evidence regarding the role of CD8dim NKT % T cells in the risk of severe COVID-19 remains elusive, thus our findings elucidate a novel causal link between these cells and the disease severity. Related studies have investigated the expression of cytotoxic molecules related to CD8 + CD4- NKT cells, with granzyme B, IFN-γ and tumor necrosis factor-α (TNF-α) showing the highest levels.^[84,85]^ IFN-γ can promote the polarization of macrophages to macrophage M1 and macrophage M1 is considered to have a proinflammatory role in COVID-19.^[13]^ Related studies have shown that compared with macrophage M2, macrophage M1 is more susceptible to virus invasion and has inducible nitric oxide synthase (iNOS) activity that converts phosphate and arginine to citrulline and NO.^[13]^ In the macrophages, IFN-γ and TNF-α synergistically signal to the Janus kinase-activated signal transducer and activator of the transcription 1 (JAK-STAT1) axis, inducing the expression of IFN regulatory factor 1 and enhancing iNOS expression activates an inflammatory cell death that causes cytokine storm called PANoptosis.^[76]^ After administration of TNF-α and IFN-γ, mice developed fatal shock with physiological symptoms consistent with those seen in patients with severe COVID-19, including multiple organ damage and dysfunction.^[90]^ Based on the above evidence, we suggest that CD8 + CD4- NKT may promote the polarization of macrophages to the M1 direction of macrophages through the above pathways and induce the corresponding macrophages to PANoptosis by enhancing the activity expression of iNOS, leading to severe cytokine storm and the aggravation of COVID-19. The active expression of iNOS in this process is equivalent to the degree of PANoptosis induced by CD8 + CD4- NKT in macrophages. PANoptosis would promote more cytokines released, spiral develop life-threatening cytokine storm, and damage to host tissues and organs nominal.^[5,90]^ Assuming that the total effect in the mediation MR analysis is accurately represented, the mediating pathway described above provides a possible explanation for how CD8dim NKT % T cells can be prevented from reducing the risk of severe COVID-19. Based on the associated molecular mechanisms, combined inhibition of IFN-γ and TNF-α, along with JAK-STAT pathway inhibitors and iNOS inhibitors, presents a novel therapeutic approach for the prevention and treatment of severe COVID-19. Preclinical studies have shown that the concurrent inhibition of IFN-γ and TNF-α signaling pathways can diminish the severity of PANoptosis-driven cytokine storms.^[5,90,91]^ The JAK1-JAK2 inhibitor Baricitinib has demonstrated efficacy in reducing the recovery time for hospitalized COVID-19 patients.^[92]^

This study aims to investigate the causal interplay among circulating immune cells, blood metabolites, and severe COVID-19, and to elucidate the role of blood metabolites-mediated circulating immune cells in disease onset and progression. Through bidirectional MR analysis, we establish a unidirectional causal relationship between circulating immune cells and severe COVID-19, mitigating the potential for reverse causation and the effects of confounding factors. Sensitivity analysis ensured the robustness of our results, and the results of mediation tests showed significantly higher statistical power. Ultimately, SNPs (*P* < 1 × 10^-5^) directly associated with the outcome factors were absent in the IVs derived from each exposure factor, which meant that it would have little effect on the results of the final analysis. Our study ultimately identified 2 potential axes contributing to severe COVID-19: circulating EM DN % DN: blood Myristate (14:0)–severe COVID-19 axis; and circulating CD8dim NKT % T cell: Citrulline to phosphate ratio–severe COVID-19 axis. This discovery not only offers potential risk markers for diagnosing severe COVID-19 but also presents new therapeutic targets for the treatment of COVID-19 co-occurring with other pathogens, the prevention and treatment of severe COVID-19, offering a foundation for developing future targeted therapies. Our study, however, has certain limitations. Despite a rigorous selection process for instruental variables, the potential influence of horizontal pleiotropy on the results cannot be entirely ruled out, a challenge inherent to all MR studies. Secondly, our analysis was conducted for the European population, which may limit the global applicability of our findings.

## 5. Conclusions

This study provides genetic evidence for evaluating the causal relationship between circulating immune cells, blood metabolites, and severe COVID-19, and reveals the role of blood metabolites-mediated circulating immune cells in the occurrence and development of severe COVID-19. Circulating EM DN %DN reduces the inhibitory effect of Myristate (14:0) on severe COVID-19 by down-regulating the level of Myristate (14:0) in blood, thereby increasing the risk of severe COVID-19. Circulating CD8dim NKT% T cell improves the promoting effect of the Citrulline to phosphate ratio on severe COVID-19 by increasing the Citrulline to phosphate ratio in the blood, which increases the risk of severe COVID-19 to a certain extent, thus resisting the possible inhibitory effect of CD8dim NKT% T cell itself on severe COVID-19. At the same time, our study provides new insights into the etiology of severe COVID-19 and related preventive and targeted therapeutic strategies.

## Acknowledgments

We would like to thank all the investigators who made their GWAS data publicly available.

## Author contributions

**Conceptualization:** Ning Ai.

**Data curation:** Ning Ai, Yan Zhang, Jing Yang, Yu Zhang, Xuejing Zhao.

**Formal analysis:** Ning Ai, Jing Yang.

**Funding acquisition:** Huifen Feng.

**Methodology:** Ning Ai, Yan Zhang.

**Project administration:** Huifen Feng.

**Software:** Yu Zhang.

**Visualization:** Xuejing Zhao.

**Writing – original draft:** Ning Ai.

**Writing – review & editing:** Huifen Feng.
